# Self-management support for patients with nonspecific neck pain: a qualitative descriptive study among physiotherapists in the United Arab Emirates

**DOI:** 10.3389/fpain.2026.1769120

**Published:** 2026-06-03

**Authors:** Azza Salim Saeed Khusoun Alketbi, Nathan Hutting, Baskaran Chandrasekaran, Gopala Krishna Alaparthi, Walid Kamal Abdelbasset, Kalyana Chakravarthy Bairapareddy

**Affiliations:** 1 Physiotherapy Department, College of Health Sciences, University of Sharjah, Sharjah, United Arab Emirates; 2 Department of Occupation and Health, School of Organization and Development, HAN University of Applied Sciences, Nijmegen, Netherlands; 3 Department of Exercise and Sports Sciences, Manipal College of Health Sciences, Manipal Academy of Higher Education, Manipal, Karnataka, India; 4 Physiotherapy, Manchester Metropolitan University, Manchester, United Kingdom

**Keywords:** non-specific neck pain, physiotherapy, qualitative study, self-management, UAE

## Abstract

**Background:**

Nonspecific neck pain poses significant morbidity and disability affecting physical, mental and social well-being. Though imperative and often recommended as a part of outpatient physiotherapy services, self-management practices remain less frequent in the United Arab Emirates due to unique cultural practices and limited training among physiotherapists in delivering self-management practices. Hence there is a need to evaluate self-management practices recommended by physiotherapists managing the patients with non-specific neck pain.

**Methods:**

A qualitative study was conducted through an online interview with open-ended questions among 16 physiotherapists in the United Arab Emirates (UAE). The interview was audio recorded via Zoom and transcribed appropriately. The quotes were reviewed and coded under themes by two independent reviewers using thematic analysis.

**Results:**

Respondents expressed the need for self-management support for patients undergoing Physiotherapy treatment for nonspecific neck pain. Patient counseling and education were commonly cited in the self-management support respondents offered. The significant aspects of education and self-care were covered by the themes in the respondents’ support. However, need to improve patient's abilities, providing more information to patients, and developing tools that support self-management were not adequately addressed by the physiotherapists in the UAE.

**Conclusion:**

Self-management support for patients with nonspecific neck pain is not optimally addressed by physiotherapists. Future research should focus on developing strategies for enhancing self-management support provided by physiotherapists to patients with non-specific neck pain.

## 1 Introduction

Non-specific neck discomfort is a leading cause of disability, with an annual prevalence ranging from 15% to 50% (1). Self-management refers to the ability of the individual with chronic diseases to manage symptoms, cope with the associated conditions and maintain treatment suggested by health professions at an optimal level (2). While self-management emphasizes enhancing self-efficacy, patient education focuses on imparting disease-specific knowledge (3).

**Table 1:** Participant characteristics.

Participant ID	Gender	Age (years)	Years of experience	Workplace setting	Institution type	Highest qualification	Specialty training
P1	Male	47	18	Outpatient clinic	Private hospital	Bachelor's	Musculoskeletal
P2	Male	35	10	Hospital	Government	Master's	Musculoskeletal
P3	Female	29	5	Outpatient clinic	Private clinic	Bachelor's	Musculoskeletal
P4	Female	28	4	Hospital	Private hospital	Bachelor's	Musculoskeletal
P5	Female	30	8	Outpatient clinic	Private clinic	Master's	Musculoskeletal
P6	Female	26	4	Hospital	Government	Bachelor's	Musculoskeletal
P7	Female	27	4	Outpatient clinic	Private clinic	Bachelor's	Musculoskeletal
P8	Female	37	13	Hospital	Private hospital	Master's	Musculoskeletal
P9	Female	27	3	Outpatient clinic	Private clinic	Bachelor's	Musculoskeletal
P10	Female	34	10	Hospital	Government	Master's	Musculoskeletal
P11	Female	38	10	Outpatient clinic	Private clinic	Bachelor's	Musculoskeletal
P12	Female	28	3	Hospital	Private hospital	Bachelor's	Musculoskeletal
P13	Female	35	10	Outpatient clinic	Private clinic	Master's	Musculoskeletal
P14	Female	25	1	Hospital	Government	Bachelor's	Musculoskeletal
P15	Female	26	5	Outpatient clinic	Private clinic	Bachelor's	Musculoskeletal
P16	Female	53	13	Hospital	Private hospital	Master's	Musculoskeletal

**Table 2:** Illustrative quotations aligned with themes and subthemes.

Theme	Subtheme	Participant ID	Illustrative quotation
Physiotherapy management approaches	Passive treatment approaches	P3	“For pain relief, I use hot pack, TENS, and ultrasound.”
		P3	“I am using gliding and soft tissue mobilization.”
	Active treatment approaches	P1	“After proper assessment, I use stretching, strengthening exercises, advice, and postural correction.”
		P4	“I provide my patients with advice related to lifestyle modifications.”
Self-management support strategies	Patient education and advice	P2	“Educating patients and simplifying advice are important.”
		P5	“For me, patient education is very important.”
	Exercise-based self-management	P7	“I ask my patients to perform the exercises in front of me to ensure they are doing it properly.”
	Self-monitoring and assessment	P6	“I use VAS for pain assessment.”
		P6	“My assessment includes ROM, muscle strength, flexibility, and neck disability index.”
	Support tools and follow-up	P10	“I provide leaflets and follow up with patients using platforms to demonstrate exercises.”
		P7	“I support my patients with instructions and videos to make exercises easy to follow.”
Understanding of self-management	Self-management as therapist-directed	P8	“Self-management is all about patients following the therapist's recommendations.”
		P9	“Self-management is a home program.”
	Self-management as independent management	P9	“Self-management is managing pain by the patients themselves.”
Professional role and training needs	Role of physiotherapist	P11	“The physiotherapist plays a critical role in self-management support.”
		P11	“We have a major role in helping patients achieve long-term outcomes.”
	Training needs and knowledge gaps	P12	“We need more knowledge about self-management skills.”
		P13	“I need more information about causes of non-specific neck pain to support patients.”
	Variation in perceived need	P14	“No need for additional knowledge to provide physiotherapy treatment.”

Although patient education and self-management support are related, they represent distinct constructs within physiotherapy practice. Routine physiotherapy management typically includes therapist-driven interventions such as manual therapy, passive modalities (e.g., electrotherapy), and supervised exercise prescription. In contrast, self-management support extends beyond clinician-directed treatment and focuses on enabling patients to actively manage their condition through the development of self-efficacy, goal setting, problem-solving abilities, symptom monitoring, and behavioral adaptation strategies (2, 4, 3). This distinction is central to contemporary biopsychosocial models of care, where patients are encouraged to take an active role in managing chronic musculoskeletal conditions. Clarifying this difference is important for interpreting physiotherapy practices related to self-management support. A recent meta-analysis on self-management for patients with non-specific neck pain found that certain educational programs may be ineffective. The authors caution that such programs might lead patients with chronic pain to concentrate more on their discomfort, potentially exacerbating their condition (5). Consequently, there is a shift towards biopsychosocial education, which includes pain management techniques (6). Some educational initiatives have been effective in preventing back and neck pain (7).

A randomized controlled trial conducted at the University of Granada compared the effects of a structured self-management program with routine physiotherapy care that primarily included joint mobilization techniques. Previous studies have demonstrated that integrating self-management strategies into physiotherapy care may improve patients' ability to manage chronic neck pain in daily life (8). Self-management programs typically include components such as patient education, symptom monitoring, problem-solving, behavioral modification, and promotion of healthy lifestyle practices (4, 3). Educational interventions may also incorporate practical guidance on posture, ergonomics, activity modification, and exercise-based self-management strategies to support independent management (9). Furthermore, evidence suggests that combining self-management approaches with conventional physiotherapy can lead to significant improvements in disability, pain, quality of life, and fear-avoidance beliefs (10). These findings are supported by qualitative research demonstrating the perceived value of self-management support strategies among physiotherapists managing musculoskeletal conditions (11, 12).

According to Global Burden of Disease (1990–2019), neck pain was found to be a significant contributing factor for age adjusted disability and years lost to disability (13). Population-based studies have also identified several demographic, occupational, and lifestyle-related factors associated with neck pain prevalence and development (20, 22, 23). Furthermore, psychological and sensorimotor variables have been shown to influence pain sensitivity and symptom severity in chronic musculoskeletal conditions (19). However, there is a lack of evidence in physiotherapist practices in delivering self-management education for patients with non-specific neck pain. We conducted a qualitative online interview that aimed to explore the self-management educational practices among physiotherapists handling non-specific neck pain. We speculate that unique cultural and social norms, heterogeneous physiotherapy practices and differences in communication practices compared to Western healthcare systems may pose barriers to the self- management education to the patients with non-specific neck pain.

## 2 Methodology

### 2.1 Study design

This study adopted a qualitative descriptive design using semi-structured interviews to explore physiotherapists’ knowledge, attitudes, and perceptions regarding the provision of self-management support for patients with non-specific neck pain. A qualitative descriptive approach was selected to generate practice-oriented insights grounded in participants’ real-world clinical experiences. The study was conducted between February and July 2021. Ethical approval was obtained from the Research Ethics Committee of the University of Sharjah (REC-21-05-05-02-S), and all procedures were conducted in accordance with the Declaration of Helsinki.

### 2.2 Participants and recruitment

A purposive sampling strategy was employed to recruit participants through the UAE Physiotherapy Association. This approach was used to ensure inclusion of physiotherapists with relevant clinical experience in managing patients with non-specific neck pain.

Eligible participants were:

- Licensed physiotherapists practicing in the UAE
- Actively involved in musculoskeletal rehabilitation
- Experienced in treating patients with non-specific neck pain

Participants represented a range of clinical settings, including outpatient clinics, hospitals, and private practice environments, thereby enhancing variability in perspectives.

Invitation emails describing the study purpose were distributed through the association. Recruitment and communication strategies were designed to enhance participation and response quality in line with recommendations for healthcare survey research (18). Interested participants contacted the research team and were enrolled after confirming eligibility. A response rate was not calculated due to the nature of voluntary recruitment through professional networks.

### 2.3 Sample size and data saturation

Sampling continued until thematic saturation was achieved. Thematic saturation was defined as the point at which no new codes, categories, or themes emerged from successive interviews. During the analysis process, the researchers noted that after approximately 13 interviews, no substantially new concepts were identified; however, three additional interviews were conducted to confirm saturation. Thus, a total of 16 interviews were included in the final analysis. Thematic saturation procedures were guided by previously established qualitative research recommendations (21). Saturation was determined through iterative review and discussion among the research team.

### 2.4 Data collection and interview procedure

Data were collected using semi-structured interviews conducted via Zoom, each lasting approximately 15–20 min. The interview guide was adapted from a previously published qualitative study (11) with permission from the original authors.

The adaptation process involved:

- Reviewing the original questionnaire for relevance to the UAE clinical context
- Modifying wording for clarity and cultural applicability
- Retaining core domains related to self-management support

The interview guide included open-ended questions exploring:

- Understanding of self-management
- Clinical practices related to patient education and support
- Perceived barriers and facilitators
- Training needs

Examples of questions included:

- “How do you support patients in managing their neck pain independently?”
- “What strategies do you use to promote self-management?”
- “What challenges do you face when implementing self-management approaches?”

The interview guide was reviewed by the research team for content relevance prior to data collection. Interviews were conducted by trained researchers with experience in qualitative interviewing and musculoskeletal physiotherapy. All participants provided informed consent prior to participation, including consent for audio recording.

### 2.5 Data analysis

Data were analyzed using Braun and Clarke's six-phase thematic analysis framework. First, the researchers familiarized themselves with the data through repeated reading of the interview transcripts. Initial codes were then generated independently by two researchers, followed by grouping related codes to identify candidate themes. These themes were subsequently reviewed, refined, and discussed until consensus was reached, after which themes were clearly defined and named. The final analytic report was developed using these themes and supported by illustrative participant quotations. A semi-deductive coding approach was employed, combining concepts informed by the interview guide with inductively derived themes emerging from the data. Coding was conducted independently by two researchers, and any disagreements were resolved through discussion and consensus. To enhance the rigor of analysis, strategies such as researcher triangulation, maintaining an audit trail, and reflexive discussions were used to strengthen the credibility and dependability of the analysis.

## 3 Results

A total of 16 physiotherapists participated in the study. Participant demographic and professional characteristics are presented in Table 1. Participants had a mean age of 32.75 years (range: 25–53 years) and an average of 7.56 years of clinical experience managing patients with non-specific neck pain. The majority were female and worked primarily in musculoskeletal practice settings.

Thematic analysis resulted in four higher-order categories. Illustrative quotations supporting the identified themes and subthemes are presented in Table 2:

1. Physiotherapy management approaches
2. Self-management support strategies
3. Understanding of self-management
4. Professional role and training needs

These categories were developed to reduce conceptual overlap and provide a clearer distinction between therapist-driven interventions and patient-centered self-management support.

## 4 Physiotherapy management approaches

### 4.1 Passive treatment approaches

Participants frequently described the use of passive treatment modalities as part of their clinical practice, including soft tissue mobilization, joint mobilization, electrotherapy, and other pain-relieving techniques. These approaches were primarily therapist-administered and aimed at symptom relief.

“For pain relief, I use hot packs, TENS, and ultrasound.”

“I am using gliding and soft tissue mobilization.”

These approaches represent routine therapist-driven interventions and were distinguished from self-management strategies, which emphasize patient autonomy and independent management.

### 4.2 Active treatment approaches

Active treatment strategies were widely reported and included exercise prescription, posture correction, and lifestyle advice. Participants emphasized improving body mechanics and encouraging physical activity.

“I provide my patients with advice related to lifestyle modifications.”

“I teach correct sitting, standing, and lifting techniques.”

Exercise-based interventions, including stretching and strengthening programs, were commonly used to improve function and reduce symptoms.

## 5 Self-management support strategies

### 5.1 Patient education and advice

Patient education emerged as a dominant strategy used by physiotherapists. Participants highlighted educating patients about their condition, pain triggers, and exercise techniques as essential components of care.

“Educating patients and simplifying advice are important.”

“I give importance to learning exercises by practice and educating patients on how to do it.”

Education was often tailored to individual patient needs and focused on improving adherence and understanding.

### 5.2 Exercise-based self-management

Participants reported encouraging patients to perform prescribed exercises independently at home, reinforcing exercise adherence as a key component of self-management.

“I always ensure that patients practice the exercises under my guidance before doing them independently.”

However, these strategies were often closely linked to therapist-prescribed programs rather than independent patient-driven management.

### 5.3 Self-monitoring and support tools

Some physiotherapists reported incorporating self-monitoring strategies and tools, such as pain scales and outcome measures, to support patient involvement.

“I use VAS for pain assessment and monitor progress.”

In addition, participants described using educational materials and digital tools:

“I provide leaflets and videos to make it easy for patients to follow exercises.”

“I follow up with patients and provide support through phone calls.”

These approaches aimed to reinforce adherence and engagement beyond the clinical setting.

## 6 Understanding of self-management

Participants demonstrated variability in their conceptual understanding of self-management. Some defined it as adherence to prescribed exercises or therapist instructions:

“Self-management is following the therapist’s recommendations.”

“Self-management is a home program.”

Others described broader interpretations involving independent management and lifestyle adaptation:

“Self-management is managing pain through daily activities and lifestyle.”

This variation indicates differing levels of alignment with established self-management frameworks.

## 7 Professional role and training needs

### 7.1 Role of physiotherapists in self-management

All participants acknowledged the important role of physiotherapists in supporting patients with non-specific neck pain.

“The physiotherapist plays a critical role in self-management support.”

“We have a major role in helping patients achieve long-term outcomes.”

The role was often described in relation to providing treatment, education, and guidance.

### 7.2 Knowledge and training needs

Many participants expressed the need for additional training and resources to effectively support self-management.

“We need more knowledge about self-management skills.”

“We need continuous development programs based on research evidence.”

Some participants also highlighted the importance of understanding psychological aspects of pain management.

However, a minority felt confident in their existing skills:

“I don’t think I need additional knowledge.”

Participants demonstrated varied interpretations of self-management, ranging from home exercise programs and adherence to therapist recommendations to broader lifestyle modifications, indicating variability in conceptual understanding. While respondents frequently described patient education, exercise instruction, and advice, these were interpreted separately from broader self-management support. Comprehensive self-management support was considered to include transferable skills such as symptom monitoring, self-regulation, problem-solving, and confidence-building, which were less consistently described by participants.

## 8 Discussion

This qualitative study explored physiotherapists’ perspectives on self-management support for patients with non-specific neck pain within the United Arab Emirates. The findings indicate that while participants recognized the importance of self-management and acknowledged their role in supporting it, their descriptions of practice were predominantly centered on health education, exercise prescription, and therapist-led interventions. Although participants emphasized physical activity, posture correction, and exercise adherence as key strategies, these approaches largely reflect routine physiotherapy management rather than comprehensive self-management support. Similar observations have been reported in previous studies, where physiotherapists often equate patient education and exercise prescription with self-management, despite the broader conceptual scope of self-management frameworks (11, 3).

An important finding of this study is the conceptual overlap between routine physiotherapy interventions and self-management support strategies. Participants frequently reported passive modalities, manual therapy, and supervised exercise as components of self-management; however, these interventions are primarily therapist-driven and do not necessarily promote patient autonomy. In contrast, self-management support involves equipping patients with transferable skills such as goal-setting, self-monitoring, problem-solving, and confidence-building to enable independent management of their condition (4, 3). This discrepancy highlights a gap between theoretical models of self-management and their practical application in clinical settings. The findings align with existing literature suggesting that physiotherapy practice continues to be dominated by biomedical and treatment-oriented approaches, with limited integration of behavioral and psychosocial strategies (14, 11). Similar concerns have been raised regarding physiotherapists' confidence in assessing psychosocial dimensions of pain and implementing person-centered care approaches in musculoskeletal rehabilitation (24, 25).

The results further indicate that physiotherapists commonly prioritize patient education and advice, focusing on posture, ergonomics, and exercise techniques. While education is an essential component of care, it alone does not constitute effective self-management support. Comprehensive self-management requires fostering patients’ ability to take an active role in their care, develop coping strategies, and adapt behaviors in response to symptoms (4). The predominance of education-focused approaches observed in this study suggests that self-management is often interpreted narrowly, which may limit its effectiveness in improving long-term outcomes. This interpretation is supported by previous research indicating that educational interventions without behavioral and psychological components may have limited impact on chronic musculoskeletal pain management (15, 3).

Another key finding is the limited integration of psychosocial and behavioral components in physiotherapy practice. Although some participants acknowledged the importance of coping strategies and patient confidence, these aspects were not consistently emphasized across responses. The dominance of biomechanical approaches, including posture correction and exercise prescription, reflects a continued reliance on biomedical models of care. This is consistent with previous studies showing that physiotherapists often underutilize psychosocial interventions and may lack confidence or training in addressing behavioral aspects of chronic pain (14, 16, 17). The absence of structured behavioral strategies in participants’ responses suggests a need to enhance training in biopsychosocial approaches to support effective self-management. This is particularly important because psychological distress, pain hypersensitivity, and psychosocial factors are increasingly recognized as major contributors to chronic musculoskeletal pain experiences (19, 25).

Contextual factors specific to the UAE may also influence the delivery of self-management support. Participants highlighted the need for additional knowledge, tools, and structured resources to support patients, which may reflect variability in training, clinical guidelines, and healthcare delivery systems. Cultural expectations, patient preferences for therapist-led care, and time constraints in clinical practice may further limit the adoption of patient-centered self-management approaches. These findings suggest that implementation of self-management strategies requires not only individual clinician training but also system-level support, including standardized protocols, educational resources, and integration of self-management frameworks into routine care. Importantly, these findings should be interpreted cautiously, as not all participants demonstrated limited knowledge; rather, there was variability in understanding and practice, with some physiotherapists expressing confidence in their current approaches while others identified areas for further development.

The findings have important implications for physiotherapy practice and education. There is a need to move beyond a predominantly biomechanical approach toward a more comprehensive biopsychosocial model that integrates behavioral and psychological strategies into routine care. Training programs should emphasize the development of skills related to behavior change, communication, and patient empowerment. Strengthening person-centered physiotherapy practice and improving clinicians' ability to evaluate psychosocial status may further enhance the effectiveness of self-management interventions (24, 25). Additionally, clinical tools and frameworks that support assessment and delivery of self-management interventions may enhance implementation in practice.

This study has several strengths. The use of qualitative interviews allowed for in-depth exploration of clinicians’ perspectives, providing insights that may not be captured through quantitative methods. Independent coding by multiple researchers and consensus-based theme development strengthened the rigor of the analysis and enhanced credibility.

However, several limitations should be considered. The relatively small purposive sample may limit the transferability of findings, and recruitment through a professional association may introduce selection bias. The reliance on self-reported practices may not fully reflect actual clinical behavior. Additionally, although efforts were made to ensure analytical rigor, the absence of member checking may limit confirmability. Future research should explore patient perspectives and evaluate the effectiveness of structured self-management interventions in clinical settings.

### 8.1 Clinical implications and future research

Most respondents in this study supported patient education and self-management. Physiotherapists should emphasize key self-management abilities while offering self-management support. Additionally, physiotherapists might benefit from self-management assistance that incorporates a biopsychosocial orientation, and self-management support should emphasize skills that can be used across disciplines (3). In the UAE, instructional programs might include a general training program for self-management support and training physiotherapists in managing various musculoskeletal conditions.

Future studies should examine the effects of including self-management skills in routine treatment plans offered by physiotherapists and other healthcare professionals. Future research should also focus on creating tools to aid healthcare practitioners in assessing patient capacities for self-management.

## 9 Conclusion

Physiotherapists in this study recognized the importance of supporting self-management for patients with non-specific neck pain; however, support was often focused on education and routine treatment advice rather than broader self-management competencies. Greater emphasis on biopsychosocial approaches, clinician training, and practical tools may strengthen delivery of self-management support in physiotherapy practice.

## Data Availability

The original contributions presented in the study are included in the article/Supplementary Material, further inquiries can be directed to the corresponding author.

## References

[1] Munk N Nemati D Benjamin EV Davies A Shue S Bair MJ. Trigger point self-care for chronic neck pain: pilot and feasibility. Adv Integr Med. (2021) 8(1):9–16.

[2] Barlow J Wright C Sheasby J Turner A Hainsworth J. Self-management approaches for people with chronic conditions: a review. Patient Educ Couns. (2002) 48(2):177–87. 10.1016/S0738-3991(02)00032-0 12401421

[3] McGowan P. Self-management education and support in chronic disease management. Prim Care Clin Off Pract. (2012) 39(2):307–25. 10.1016/j.pop.2012.03.005 22608868

[4] Jonkman NH Schuurmans MJ Jaarsma T Shortridge-Baggett LM Hoes AW Trappenburg JCA. Self-management interventions: proposal and validation of a new operational definition. J Clin Epidemiol. (2016) 80:34–42. 10.1016/j.jclinepi.2016.08.001 27531245

[5] Martinez-Calderon J Flores-Cortes M Morales-Asencio JM Luque-Suarez A. Psychological factors involved in the onset and/or persistence of musculoskeletal pain: an umbrella review of systematic reviews and meta-analyses of prospective cohort studies. Clin J Pain. (2020) 36(8):626–37. 10.1097/AJP.0000000000000838 32379072

[6] Andias R Silva AG. Psychosocial variables and sleep associated with neck pain in adolescents: a systematic review. Phys Occup Ther Pediatr. (2020) 40(2):168–91. 10.1080/01942638.2019.1647328 31364900

[7] Ainpradub K Sitthipornvorakul E Janwantanakul P van der Beek A. Effect of education on non-specific neck and low back pain: a systematic review of randomized controlled trials. Physiotherapy. (2015) 101:e671. 10.1016/j.physio.2015.03.3511

[8] Monaghan J Fothergill M Adams N. Challenges in the self-management of low back pain from the physiotherapist perspective. Physiotherapy. (2016) 102:e177.

[9] Côté P, Wong JJ, Sutton D, Shearer HM, Mior S, Randhawa K, et al. Management of neck pain and associated disorders: a clinical practice guideline from the Ontario Protocol for Traffic Injury Management (OPTIMa) Collaboration. *Eur Spine J.* (2016) 25(7), 2000–22. 10.1007/s00586-016-4467-7 26984876

[10] López-López L Ariza-Mateos M Rodríguez-Torres J Cabrera-Martos I Granados-Santiago M Torres-Sánchez I. Results of a Self-management program Added to Standard Physical Therapy for Chronic Neck Pain. Amsterdam: Patient Education and Counseling (2020). 10.1016/j.pec.2020.11.014 33246873

[11] Hutting N Oswald W Staal J Heerkens Y. Self-management support for non-specific low back pain: a qualitative survey among physiotherapists and exercise therapists. Musculoskelet Sci Pract. (2020) 50:102269. 10.1016/j.msksp.2020.102269 33039797

[12] Mork R Falkenberg HK Fostervold KI Thorud HMS. Discomfort glare and psychological stress during computer work: subjective responses and associations between neck pain and trapezius muscle blood flow. Int Arch Occup Environ Health. (2020) 93(1):29–42. 10.1007/s00420-019-01457-w 31286223

[13] Ahangar-Sirous R, Alizadeh M, Nejadghaderi SA, Noori M, Khabbazi A, Sullman MJ, et al. The burden of neck pain in the Middle East and North Africa region. Heliyon. (2023) 9(11). Available online at: https://www.cell.com/heliyon/fulltext/S2405-8440(23)08504-3 10.1016/j.heliyon.2023.e21296 PMC10643100 38027849

[14] Alexanders J Anderson A Henderson S. Musculoskeletal physiotherapists’ use of psychological interventions: a systematic review of therapist perceptions and practice. Physiotherapy (Lond). (2015) 101(2):95–102. 10.1016/j.physio.2014.03.008 25435081

[15] Dwamena F. Patient-centered shared decision making. Perm J. (2012) 16(4):74.

[16] Brunner E Dankaerts W Meichtry A O’Sullivan K Probst M. Physical therapists’ ability to identify psychological factors and their self-reported competence to manage chronic low back pain. Phys Ther. (2018) 98(6):471–9. 10.1093/ptj/pzy012 29385524

[17] Setchell J Costa N Ferreira M Makovey J Nielsen M Hodges PW. Individual explanations for their persistent or recurrent low back pain: a cross-sectional survey. BMC Musculoskelet Disord. (2017) 18(1):1–9. 10.1186/s12891-017-1831-7 29149847 PMC5693501

[18] Funkhouser E Vellala K Baltuck C Cacciato R Durand E McEdward D. Survey methods to optimize response rate in national dental practice-based research network. Eval Health Prof. (2016) 40(3):332–58. 10.1177/0163278715625738 26755526 PMC5002250

[19] Garrigós-Pedrón M La Touche R Navarro-Desentre P Gracia-Naya M Segura-Ortí E. Widespread mechanical pain hypersensitivity in patients with chronic migraine and temporomandibular disorders: relationship and correlation between psychological and sensorimotor variables. Acta Odontol Scand. (2019) 77(3):224–31. 10.1080/00016357.2018.1538533 30626243

[20] Genebra CVDS Maciel NMTPF Bento TPF Simeão SFAP De Vitta A. Prevalence and factors associated with neck pain: a population-based study. Braz J Phys Ther. (2017) 21(4):274–80. 10.1016/j.bjpt.2017.05.005 28602744 PMC5537482

[21] Guest G Namey E Chen M. A simple method to assess and report thematic saturation in qualitative research. PLoS One. (2020) 15(5):e0232076. 10.1371/journal.pone.0232076 32369511 PMC7200005

[22] Haldeman S Carroll L Cassidy JD. Findings from the bone and joint decade 2000 to 2010 task force on neck pain and its associated disorders. J Occup Environ Med. (2010) 52(4):424–7. 10.1097/JOM.0b013e3181d44f3b 20357682

[23] Kim R Wiest C Clark K Cook C Horn M. Identification of risk factors for first-episode neck pain: a systematic review. Musculoskelet Sci Pract. (2018) 33:77–83. 10.1016/j.msksp.2017.11.007 29197234

[24] Mudge S Stretton C Kayes N. Are physiotherapists comfortable with person-centered practice? An autoethnographic insight. Disabil Rehabil. (2014) 36(6):457–63. 10.3109/09638288.2013.797515 23713969

[25] Singla M Jones M Edwards I Kumar S. Physiotherapists’ assessment of patients’ psychosocial status: are we standing on thin ice? A qualitative descriptive study. Man Ther. (2015) 20(2):328–34. 10.1016/j.math.2014.10.004 25454686

